# Upregulation of the Tim-3/Galectin-9 Pathway of T Cell Exhaustion in Chronic Hepatitis B Virus Infection

**DOI:** 10.1371/journal.pone.0047648

**Published:** 2012-10-24

**Authors:** Gaia Nebbia, Dimitra Peppa, Anna Schurich, Pooja Khanna, Harsimran D. Singh, Yang Cheng, William Rosenberg, Geoffrey Dusheiko, Richard Gilson, Joanne ChinAleong, Patrick Kennedy, Mala K. Maini

**Affiliations:** 1 Division of Infection and Immunity, University College London, London, United Kingdom; 2 Centre for Hepatology, University College London, London, United Kingdom; 3 Centre for Sexual Health and HIV, University College London, London, United Kingdom; 4 Centre for Digestive Diseases, Institute of Cell and Molecular Science, Barts and the London School of Medicine and Dentistry, London, United Kingdom; University of Montreal, Canada

## Abstract

The S-type lectin galectin-9 binds to the negative regulatory molecule Tim-3 on T cells and induces their apoptotic deletion or functional inactivation. We investigated whether galectin-9/Tim-3 interactions contribute to the deletion and exhaustion of the antiviral T cell response in chronic hepatitis B virus infection (CHB). We found Tim-3 to be expressed on a higher percentage of CD4 and CD8 T cells from patients with CHB than healthy controls (p<0.0001) and to be enriched on activated T cells and those infiltrating the HBV-infected liver. Direct *ex vivo* examination of virus-specific CD8 T cells binding HLA-A2/peptide multimers revealed that Tim-3 was more highly upregulated on HBV-specific CD8 T cells than CMV-specific CD8 T cells or the global CD8 T cell population in patients with CHB (p<0.001) or than on HBV-specific CD8 after resolution of infection. T cells expressing Tim-3 had an impaired ability to produce IFN-γ and TNF-α upon recognition of HBV-peptides and were susceptible to galectin-9-triggered cell death *in vitro*. Galectin-9 was detectable at increased concentrations in the sera of patients with active CHB-related liver inflammation (p = 0.02) and was strongly expressed by Kupffer cells within the liver sinusoidal network. Tim-3 blockade resulted in enhanced expansion of HBV-specific CD8 T cells able to produce cytokines and mediate cytotoxicity in vitro. Blocking PD-1 in combination with Tim-3 enhanced the number of patients from whom functional antiviral responses could be recovered and/or the strength of responses, indicating that these co-inhibitory molecules play a non-redundant role in driving T cell exhaustion in CHB. Patients taking antivirals able to potently suppress HBV viraemia continued to express Tim-3 on their T cells and respond to Tim-3 blockade. In summary, both Tim-3 and galectin-9 are increased in CHB and may contribute to the inhibition and deletion of T cells as they infiltrate the HBV-infected liver.

## Introduction

Galectin-9 is one of a family of soluble β-galactoside–binding proteins that have been reported to have a multifactorial role in T cell development and homeostasis [1]. When first described, it was noted to be abundantly expressed in foetal and adult murine liver [2]. It was also found to be heavily expressed in the thymus, where it was proposed to play a role in deleting developing thymocytes [2]. Galectin-9 binds to T cell immunoglobulin and mucin domain-containing molecule (Tim-3), which was initially identified as a membrane protein on terminally differentiated T helper type 1 (Th1) T cells [3]. Tim-3/galectin-9 interactions have been found to drive death of Th1 T cells and promote peripheral tolerance [4]. More recently, Tim-3 has been recognised to also have the potential to contribute to the functional inactivation of CD8 T cells in persistent viral infections [5], [6].

The antiviral T cell response in patients with chronic HBV infection (CHB) is markedly depleted and prone to apoptosis [7]. The few residual T cells bear the hallmark of profound exhaustion, with high expression of the co-inhibitory receptors PD-1 [8] and CTLA-4 [9] and poor effector function [8], [10]. Tim-3 has been reported to mark those T cells that have progressed to advanced stages of the hierarchical impairment of effector function characteristic of T cell exhaustion, defined by loss of IFN-γ production [5], [6]. We therefore postulated that Tim-3 would be upregulated on the exhausted T cells in patients with CHB, which have often been exposed to decades of extremely high antigenic load.

**Figure 1:**
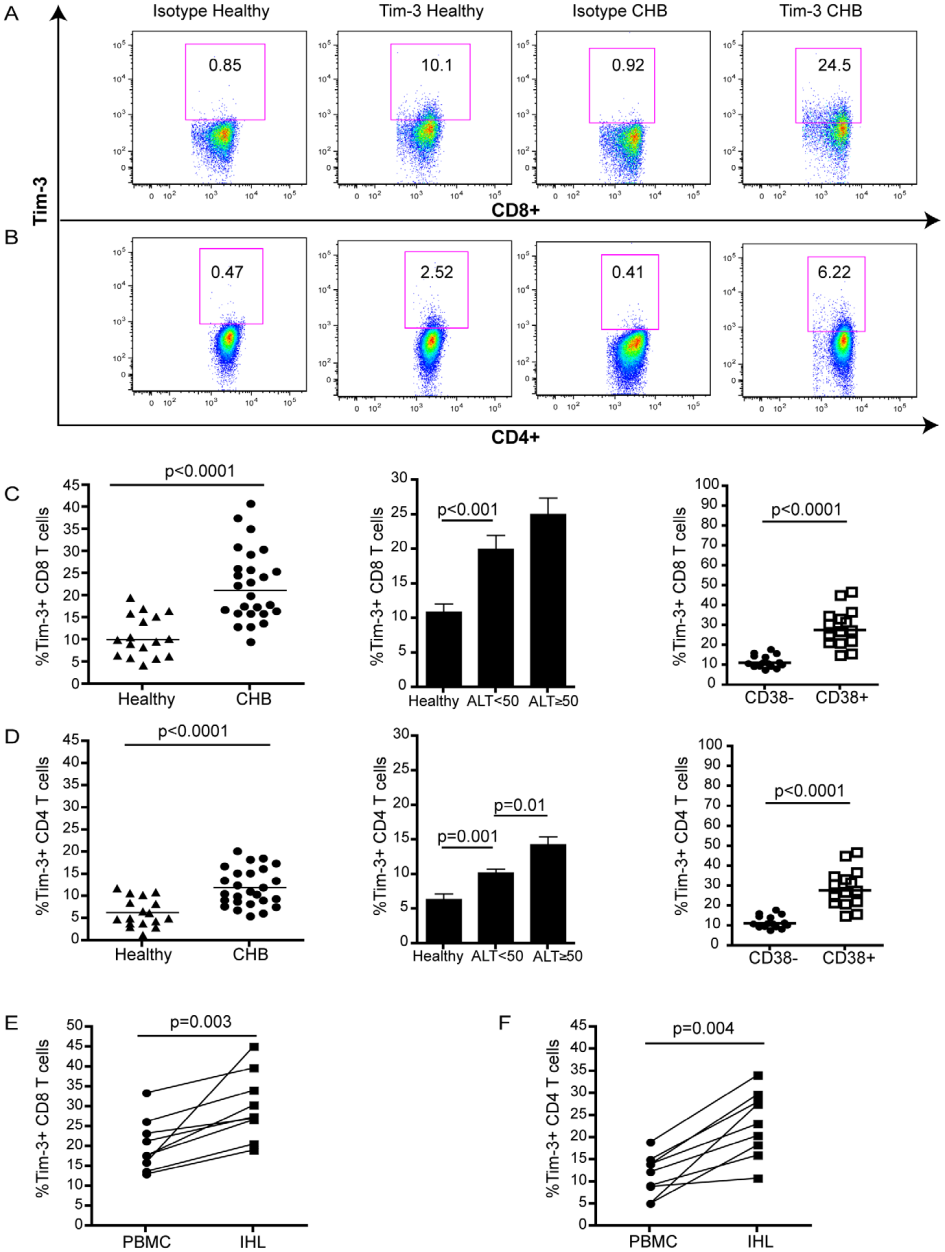
Tim-3 is up-regulated on peripheral and intrahepatic T cells in HBV infection. PBMC from 26 HBV infected and 17 uninfected subjects were stained with antibodies against CD3, CD4, CD8, CD38 and Tim-3 or an isotype-matched control Ig. Representative flow cytometry data for (a) CD8 and (b) CD4 T cells from a patient with CHB and a healthy subject. Tim-3 expression on global (c) CD8 and (d) CD4 T cells from healthy individuals and patients with CHB, shown as compiled data and stratified according to ALT level or CD38 co-expression. Expression of Tim-3 on (e) CD8 and (f) CD4 of paired PBMC and IHL from 8 patients with CHB. For all figures, statistical analyses were performed using Mann-Whitney test or the Wilcoxon rank test for paired data; only significant p values (p<0.05) are indicated. Horizontal bars denote medians.

**Table 1:** Patient clinical characteristics.

	CHB (n = 111)	Healthy (n = 20)	Resolved (n = 6)
Gender	Male = 61	Male = 9	Male = 4
Age (years)	33 (21–68)	33 (20–47)	37 (28–43)
ALT (IU/L)	44 (10–1208)	NA	NA
HBV DNA (log IU/ml)	3.6 (1.3–9.1)	NA	NA
eAntigen +	n = 31	NA	NA

* Median and range shown.

** NA not applicable.

We further hypothesised that galectin-9/Tim-3 interactions might be of particular relevance in HBV infection because it targets the liver. In a mouse model of acute graft versus host disease, CD8 T cells in the liver were noted to have particularly high Tim-3 expression; blockade of Tim-3 suggested it played an important part in the maintenance of hepatic tolerance [11]. The original description of galectin-9 being preferentially expressed in the liver and localised close to the sinusoids [2], suggested that it could be ideally positioned to promote deletion or functional inactivation of Tim-3-expressing HBV-specific CD8 T cells as they infiltrate the site of infection. In this study we report that Tim-3 is upregulated on both CD4 and CD8 T cells in CHB, particularly the intrahepatic fraction. HBV-specific T cells with impaired production of IFN-γ and TNF-α have increased Tim-3 expression and blockade results in recovery of functionally active T cells. The ligand, galectin-9, is upregulated in patients with active disease and is highly expressed by the Kupffer cell population in the hepatic sinusoids.

## Results

### Increased Tim-3 Expression on Global T Cells from Patients with CHB

In order to assess the potential role of Tim-3 in CHB, we first examined expression of the glycoprotein Tim-3 directly ex vivo by flow cytometry on PBMC from 26 CHB and 17 healthy individuals (Table 1). Consistent with previous observations [12], we found elevated frequencies of Tim-3-expressing global CD8 T cells in individuals with CHB compared to healthy controls (median of 21.2 versus 10.1 respectively, p<0.0001) (Figure 1a, c). There was a non-significant trend towards a higher frequency of Tim-3+ expressing CD8 T cells in CHB patients with high (>2000 IU/ml) versus low (<2000 IU/ml) HBV DNA (data not shown). The frequency of Tim-3+ CD8 T cells did not correlate with with eAg status (data not shown) but showed a stepwise increase with increasing liver inflammation (as assessed by ALT level, Figure 1c). Tim-3 expression was also elevated on global CD4 T cells in HBV infected individuals relative to uninfected controls (p<0.0001) and correlated with ALT (Figure 1b, d), although it was expressed at a lower overall frequency than on CD8 T cells. In keeping with this global increase in Tim-3 expression, there was an increase in T cells expressing the activation marker CD38 in CHB (Figure S1a); Tim-3 expression was enriched on this activated fraction (Figure 1c, d).

**Figure 2:**
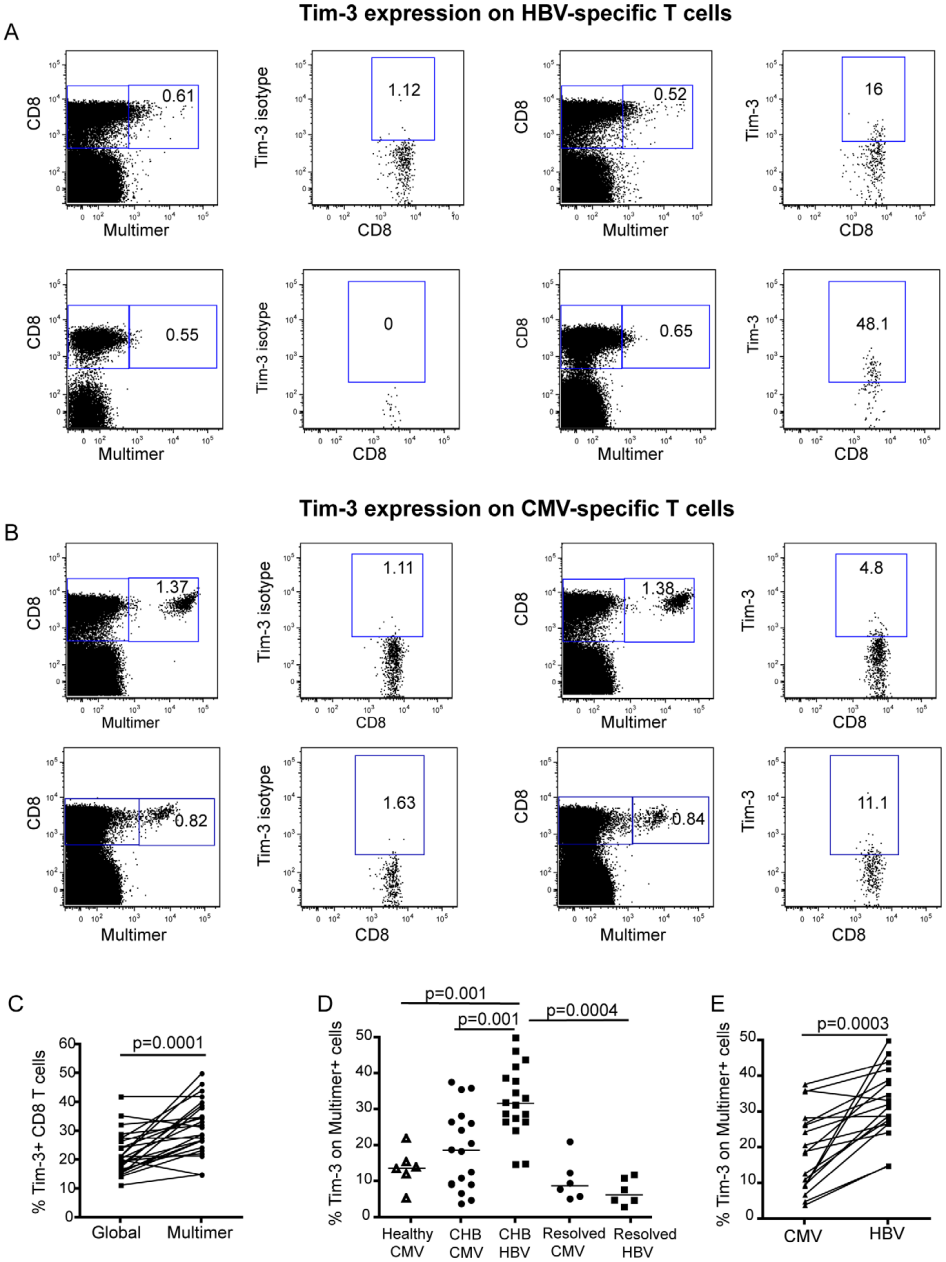
Tim-3 expression is increased on HBV-specific T cells. HLA-A2+ healthy controls and CHB patients were stained *ex vivo* with HLA-A2 multimers presenting the CMV-pp65 epitope NLVPMVATV (HLA-A2/NLVP) or a combined panel of 6 HBV-multimers (HLA-A2/core 18–27, envelope 183–191, envelope 348-, envelope 335-,polymerase 455-, polymerase 502-) and with anti-Tim-3 mAb or its isotype. Representative FACS plots from two patients with CHB showing staining for HBV (a) and CMV (b) multimers and Tim-3 expression on gated multimer–specific CD8 T cells compared to an isotype control mAb. (c) Compiled data showing the frequency of virus-specific (multimer+) and global CD8 T cells expressing Tim-3 directly *ex vivo* in 24 CHB patients. (d) Compiled data showing the frequency of HBV and CMV-specific CD8 T cells expressing Tim-3 directly *ex vivo* in healthy controls (n = 6), patients with CHB (n = 24) and patients who had resolved HBV (n = 6). (e) *Ex-vivo* staining in 17 individuals with CHB in whom paired responses could be analysed with both HBV and CMV multimers.

In order to investigate whether Tim-3-expressing T cells were enriched in the intrahepatic compartment, the site of HBV replication, we obtained paired liver biopsies and blood samples from 8 patients with CHB. We isolated intrahepatic lymphocytes (IHL) and compared the expression of Tim-3 on global CD8 and CD4 T cells from the liver and blood of the same patients. A higher frequency of Tim-3 expressing T cells was seen in the intrahepatic compartment in every case for both CD8 and CD4 T cells (p = 0.003 and p = 0.004 respectively (Figure 1e, f, Figure S1b); this may reflect enhanced induction at the site of disease or preferential liver tropism of Tim-3 expressing cells.

**Figure 3:**
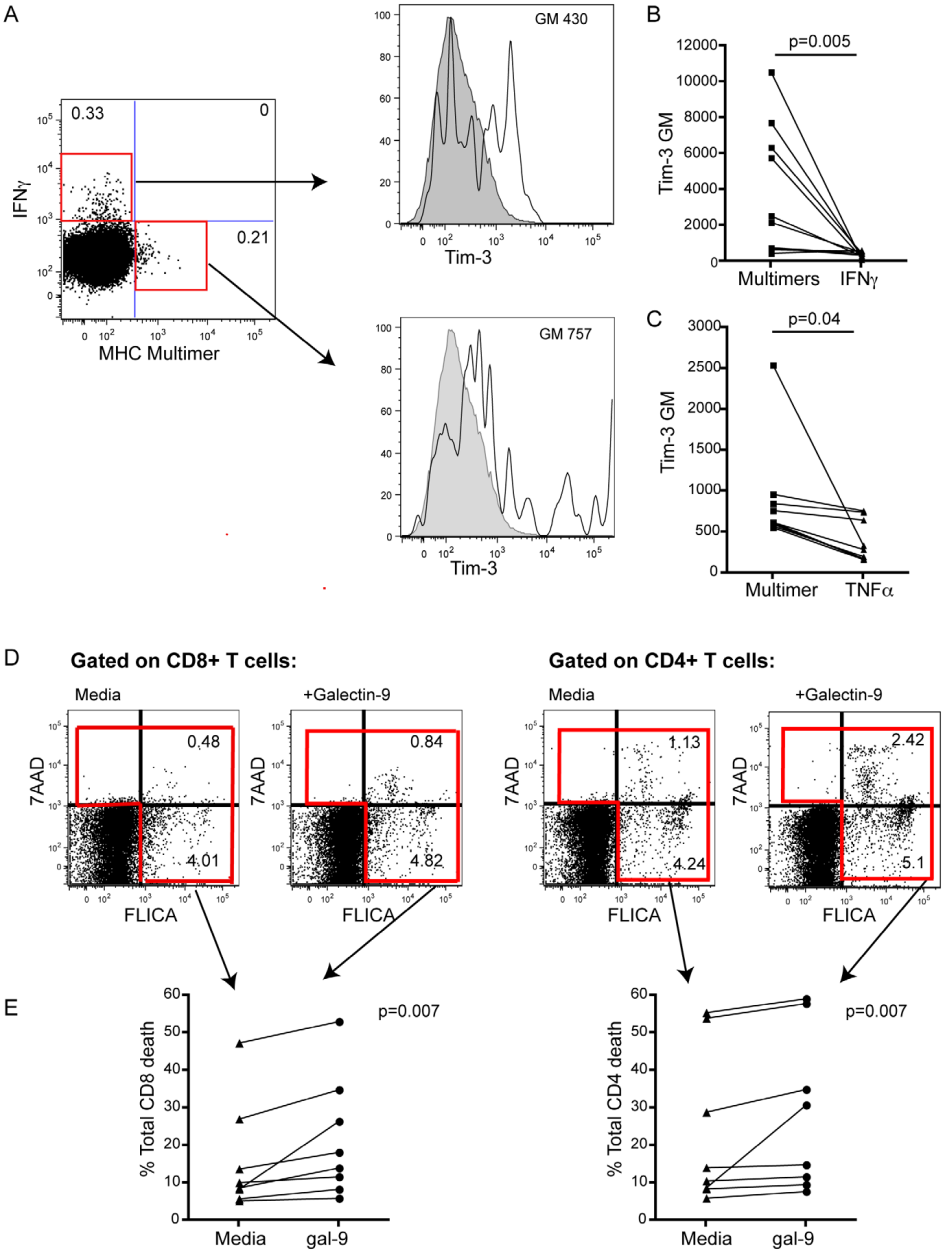
Tim-3 expressing HBV-specific T cells are dysfunctional. PBMC derived from HLA A2+ patients with CHB were stained with a panel of HLA-A2/HBV multimers and then were stimulated overnight with a pool of HBV peptides of matched specificity to the multimers, followed by intracellular staining for IFN-γor TNF-α. (a) Representative histograms showing levels of Tim-3 (black line) or isotype binding (grey shading) on CD8 T cells binding HLA-A2/HBV peptide multimers or producing IFN-γ upon encounter with HBV peptides. (b) Compiled data from 10 patients with CHB. (c) Tim-3 expression on CD8 T cells binding HLA-A2/HBV peptide multimers or producing TNFa upon stimulation with HBV peptides. (d) FACS plots and (e) summary data showing the induction of caspases (FLICA) and 7AAD in CD8 and CD4 T cells with or without the addition of galectin-9. Active caspases, indicating apoptosis, were determined using a fluorescent-labelled inhibitor of polycaspases (FAM-VAD-FMK, FLICA), and death was identified by 7AAD stain. Early apoptotic events are indicated in the lower right quadrant (FLICA+7AAD−), late apoptotic events in the right upper quadrant (FLICA+7AAD+) and necrotic cells in the left upper quadrant (7AAD+FLICA-). ‘Total death’ was estimated by summing events in these 3 quadrants.

### Up-regulation of Tim-3 on HBV-specific CD8+ T Cells with Impaired Effector Function and Apoptotic Propensity

Having observed an upregulation of Tim-3 on global T cells from chronically infected individuals, we then looked at the expression of this negative regulator on HBV-specific T cells. Using a panel of HBV and CMV HLA-A2/peptide multimers, we assessed Tim-3 expression on the surface of HBV and CMV-specific CD8 T cells detectable *ex vivo* in PBMC samples from 24 patients with CHB (Table S1), 6 patients who had resolved HBV and 6 healthy volunteers (Figure 2a, b). The frequency of HBV-specific CD8 T cells expressing Tim-3 was significantly higher than the global CD8 population in the same patients (p<0.001, Figure 2c). Tim-3 expressing HBV-specific CD8 T cells were increased in patients with CHB (regardless of VL or ALT, data not shown) compared to those who had resolved infection successfully (p<0.001, Figure 2d). We also observed significantly higher expression of Tim-3 on HBV-specific compared to CMV-specific CD8 T cells in patients with CHB (p = 0.001)(Figure 2d). For 17 patients with CHB we were able to examine paired HBV and CMV-specific CD8 T cell responses, confirming higher frequencies of Tim-3-expressing CD8 T cells directed against HBV than CMV epitopes within the same patients (p<0.001, Figure 2e). CMV-specific CD8 T cells in HBV-infected subjects showed a trend towards higher Tim-3 expression than CMV-specific CD8 T cells in resolved or healthy controls, (Figure 2d), in line with the global upregulation of Tim-3 on global T cells in CHB.

**Figure 4:**
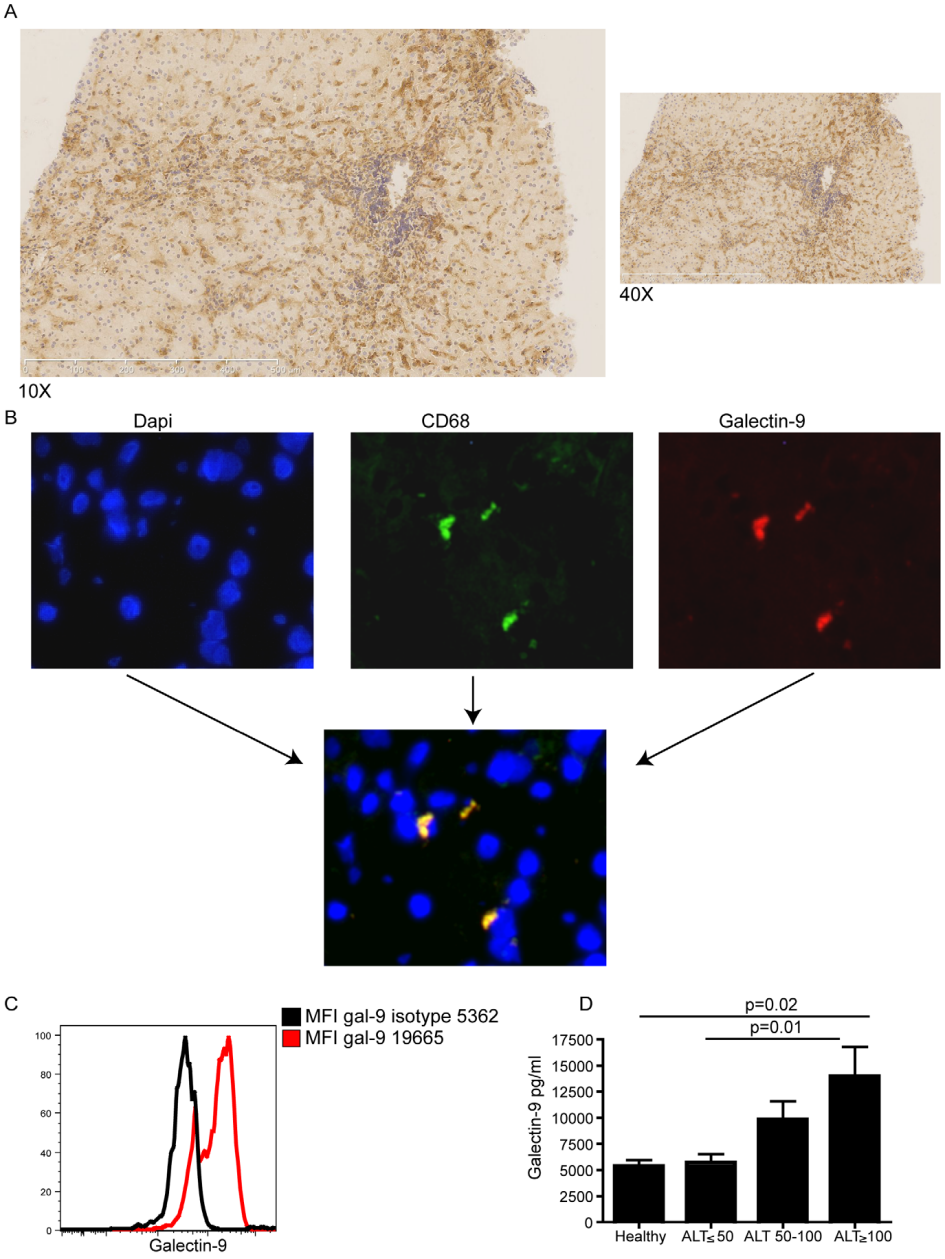
The Tim-3 ligand galectin-9 is expressed by Kupffer cells, and its secretion is increased in active CHB. Immunohistochemistry of a section from a cryopreserved CHB liver biopsy stained with a polyclonal galectin-9 antibody (shown in brown) at 10X and 40X magnification. (b**)** Immunofluorescence of a section from a cryopreserved CHB liver biopsy stained with DAPI (blue, left panel), anti-CD68 mAb (green, central panel) and galectin 9 polyclonal antibody (red, right panel). Double positive staining is indicated in yellow in the lower panel. (c) Kupffer cells were isolated from 4 liver explants and stained with CD14, CD68 and galectin-9 or its isotype. Representative histograms show galectin-9 staining on CD14+CD68+ fraction compared to isotype-matched control. (d) Galectin-9 levels were analysed in the serum of 10 healthy subjects and 42 CHB patients: 16 with ALT<50, 17 with ALT 50–100 and 9 with ALT >100 (mean and SEM shown).

**Table 2:** Clinical details of patient who underwent liver biopsy and galectin-9 quantification from serum samples.

	Sera		Histology	
	CHB (n = 34)	Healthy (n = 10)	CHB (n = 8)	Healthy (n = 5)
Gender	Male = 19	Male = 6	Male = 6	Male = 3
Age	34 (21–68)	31 (24–47)	47 (29–67)	45 (27–66)
ALT (IU/L)	53.5 (11–1208)	NA	52 (22–189)	25 (8–120)
HBV DNA (log IU/ml)	4.6 (1.4–9.12)	NA	4.4 (1.4–7.2)	NA
eAg status	eAg+ = 12	NA	eAg+ = 5	NA

* Median and range shown.

** NA not applicable.

Previous studies of patients with chronic HIV [6] and HCV [5] infection have described impaired effector function of Tim-3-expressing CD8 T cells. We therefore assessed the functionality of Tim-3+ HBV-specific CD8 T cells, focusing on their production of the cytokines IFN-γ and TNF-α, which have been shown to be able to purge HBV from infected hepatocytes [13]. PBMC were labelled with a panel of HLA-A2/peptide multimers representing well-described HLA-A2-restricted HBV epitopes, stimulated with a pool of peptides of the same specificities and stained for their ability to produce IFN-γ or TNF-α. As reported previously [14], there was a small multimer-negative population of CD8 able to produce cytokines upon stimulation with HBV peptides (Figure 3a, Figure S1c). CD8 T cells able to bind HLA-A2/HBV peptide multimers but unable to produce IFN-γ expressed higher levels of Tim-3 than those cells that retained the capacity to produce IFN-γ upon encounter with cognate peptide (p = 0.005) (Figure 3a,b, Figure S1c). Likewise, those HBV-specific responses able to produce TNF-α had reduced expression of Tim-3 (Figure 3c *).* Tim-3 expression therefore defines a population of dysfunctional T cells in the context of chronic HBV infection.

**Figure 5:**
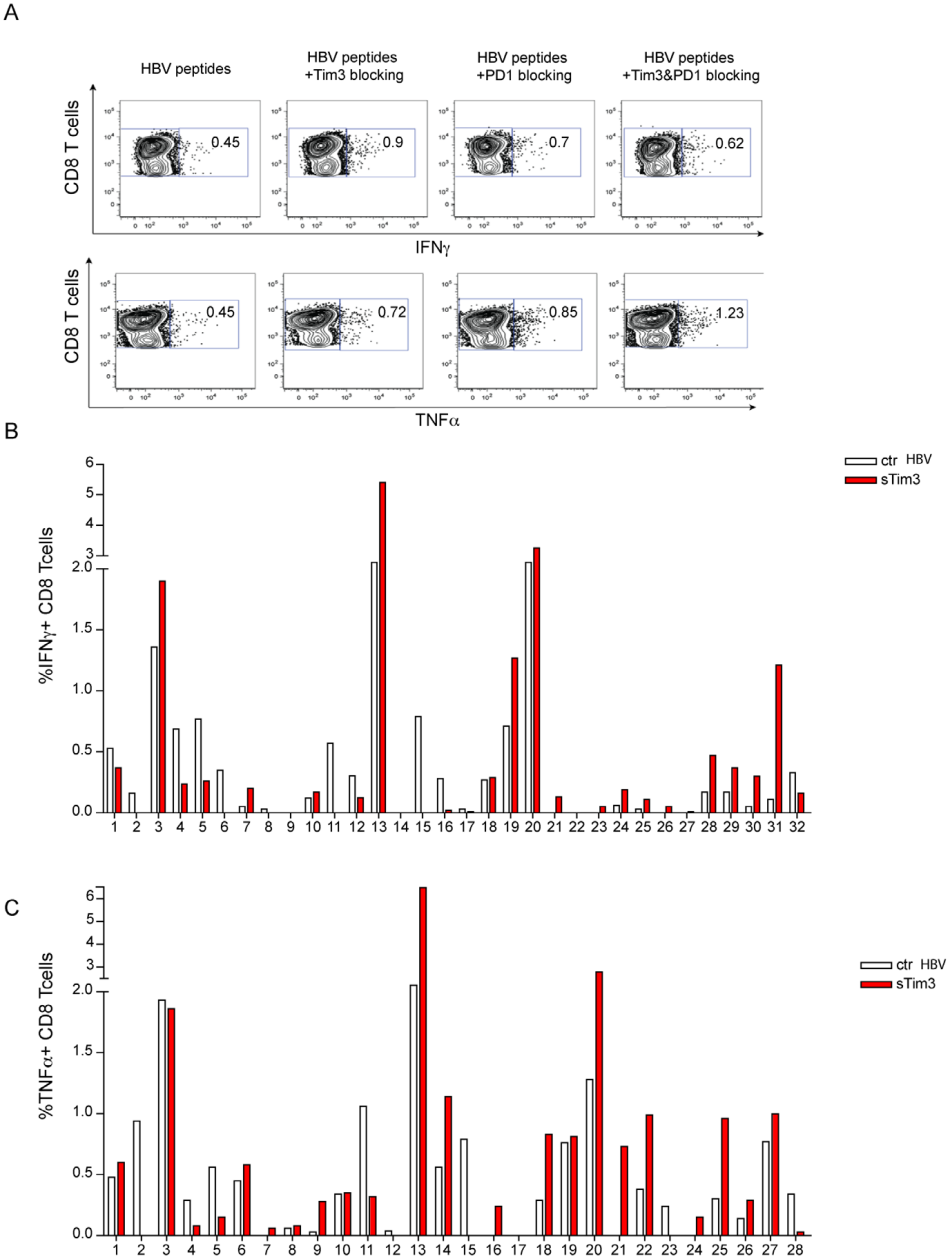
Blocking the Tim-3 pathway can increase the frequency of IFNγ and TNFα-producing HBV-specific CD8 T cells. (a) Representative dot plot showing recovery of HBV-specific CD8 T cells responses (IFN-γ, TNF-α) in a patient with CHB. PBMC were stimulated with peptides and cultured for 10 days in the presence of soluble Tim-3 FC chimera, PDL1/L2 blocking antibody or both. Summary data of the effect of blocking Tim-3 on IFN-γ (b) and TNF-α (c) production by CD8 T cells in response to HBV peptides.

Engagement of Tim-3 by its ligand, galectin-9, has also been shown to reduce IFN-γ production by inducing death of Th1 T cells [4]. We therefore evaluated the potential of this interaction to dampen immune responses through induction of cell death in the context of CHB. PBMC from patients with CHB were cultured in the presence of recombinant galectin-9 or media alone for 6 hours. A fluorescent probe able to bind active caspases (FLICA) was added for the last hour of culture and cells were then stained for the death marker 7AAD (Figure 3d). Although CD4 and CD8 T cells showed high levels of baseline apoptosis/death as reported previously in patients with CHB [7], [9], there was a variable further increase in expression of active caspases and 7AAD, suggesting galectin-9 mediated induction of both early/late apoptosis and cell death (Figure 3d, e). When the cells were co-cultured in the presence of both galectin-9 and soluble Tim-3 chimera, there was partial blocking of death induction (Figure S2a).

**Figure 6:**
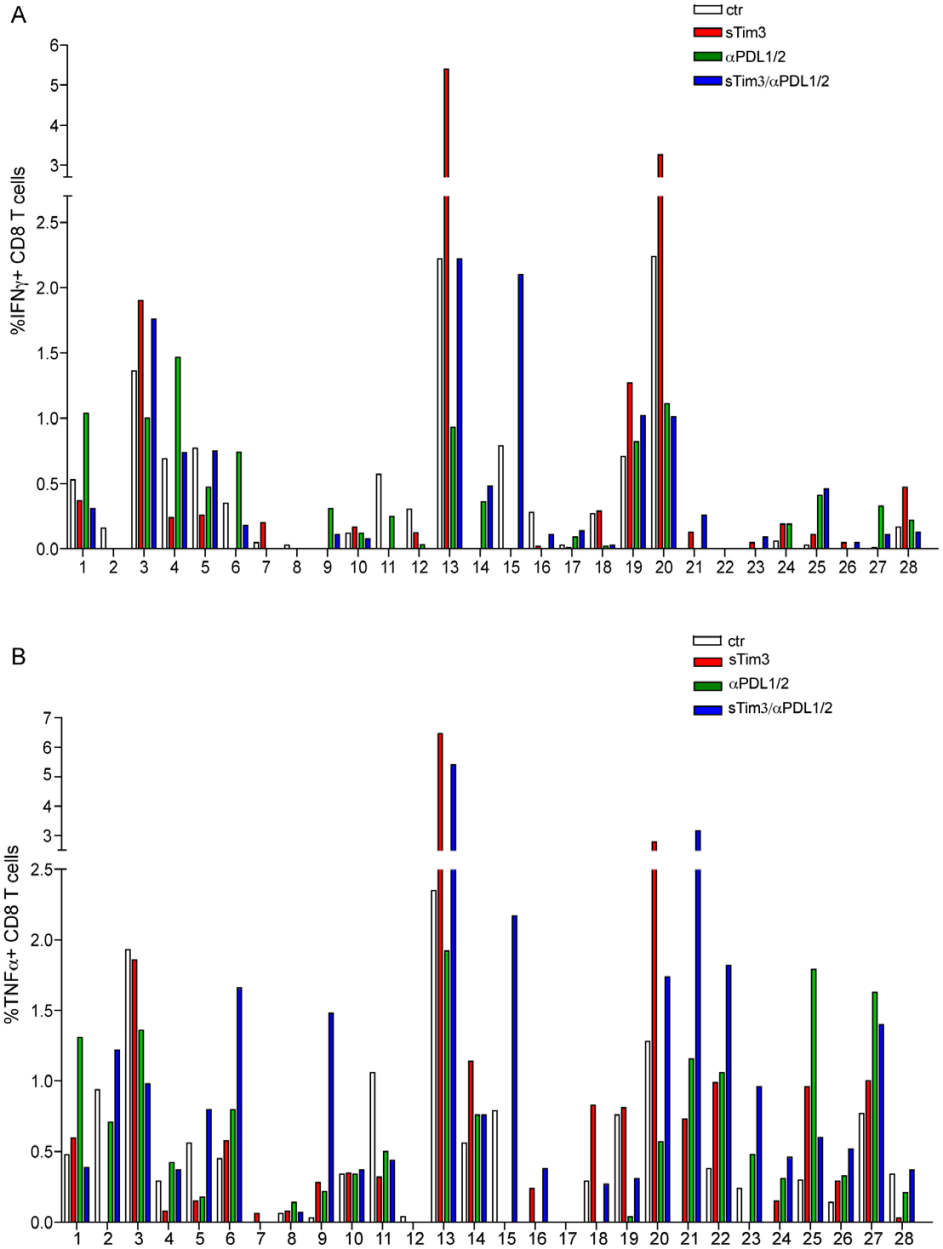
Non-redundant roles for Tim-3 and PD-L1/2 blockade in recovery of functional HBV-specific T cell responses. Summary data from 28 patients with CHB of the percent of HBV-specific CD8 T cells producing IFN-γ (a) or TNF-α (b) upon blockade of Tim-3 (red bars), PDL1/L2 (green bars) or dual Tim-3 and PDL1/L2 (blue bars), compared to stimulation with peptides without blocking (white bars).

**Figure 7:**
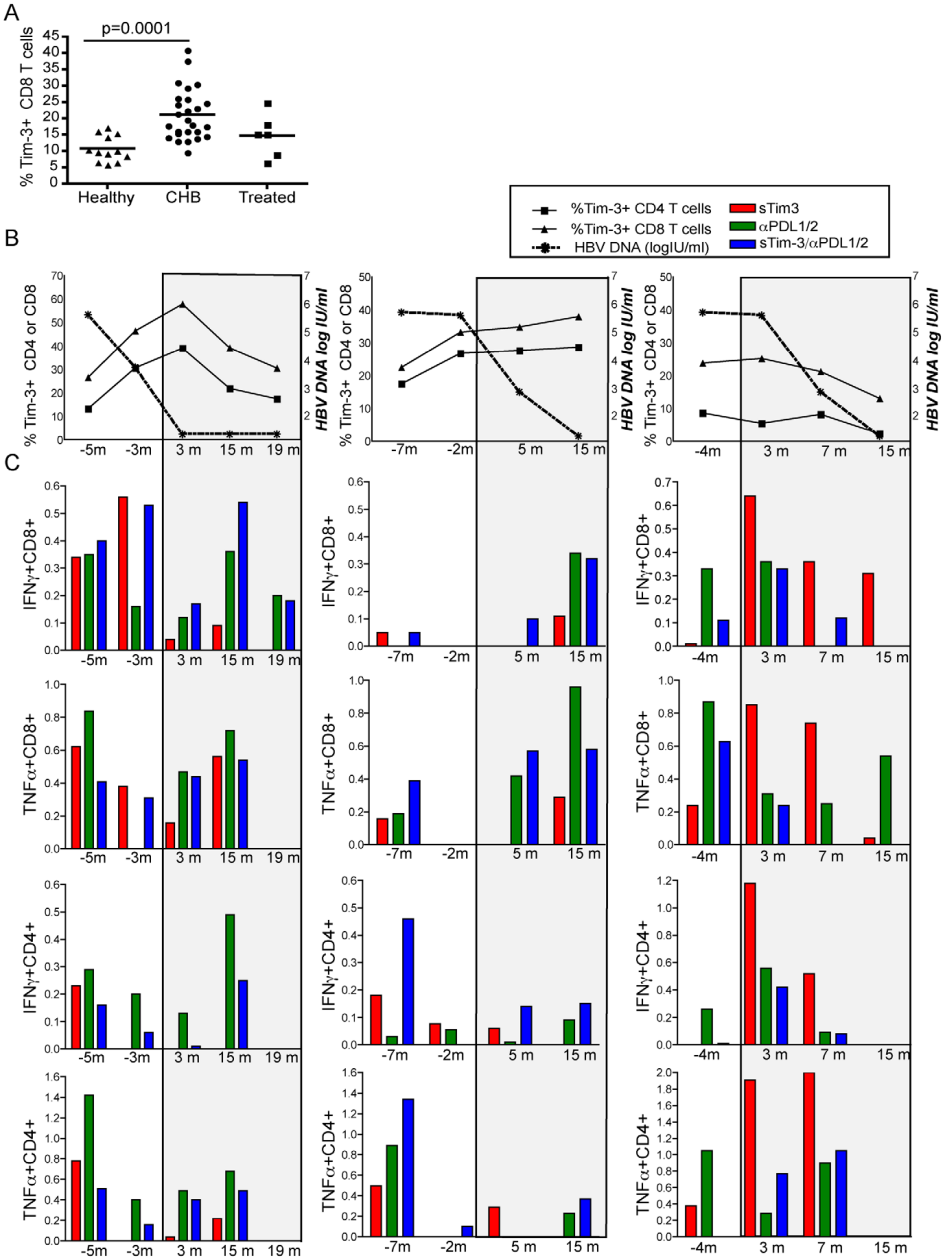
Effect of antiviral treatment on Tim-3 expression and response to Tim-3 blockade. (a) Tim-3 expression on global CD3 CD4+ T cells and CD3 CD8+ T cells. Each dot represents an individual data point (12 healthy, 26 CHB, 6 CHB on antivirals with undetectable viral load); horizontal lines represent the mean. (b) Percent of CD4 (square), CD8 (triangle) T cells expressing Tim-3 (left y axis) and HBV load circle, right y axis) plotted longitudinally for 3 patients starting antiviral therapy. (c) PBMC sampled at the indicated time points from these three individuals were stimulated for ten days with HBV OLP with control IgG, Tim-3 Fc chimera, anti-PDL1/PDL2 antibodies or both. Bars represent the % of HBV-specific CD8 T cells producing IFN-γ or TNF-α following blockade after subtracting the frequency detectable without any blockade.

**Table 3:** Clinical characteristics of patients followed up longitudinally.

Patient	Time point	HBV DNA (log IU/ml)	ALT (IU/L)	eAg status	Gender	Age
1	5 months pre-Tx	5.62	116	neg	Male	39
	3 months pre-Tx	3.77	500	neg		
	3 months post-Tx	1.40	40	neg		
	15 months post Tx	1.40	46	neg		
	19 months post-tx	1.40	47	neg		
2	7 months pre-Tx	6.15	58	pos	Male	63
	2 months pre-Tx	6.4	53	pos		
	5 months post-Tx	3.10	207	pos		
	15 months post-Tx	1.40	15	pos		
3	4 months pre-Tx	4.86	405	neg	Male	47
	3 months pre-Tx	1.40	33	neg		
	7 months post-Tx	1.40	27	neg		
	15 months post-Tx	1.40	30	neg		

### The Tim-3 Ligand Galectin-9 is Expressed by Kupffer Cells and its Secretion is Increased in Active CHB

The natural ligand for Tim-3, galectin-9, was originally reported to be preferentially expressed in the liver [2]. We therefore postulated that Tim-3-expressing HBV-specific CD8 T cells encounter their ligand as they infiltrate the liver, the site of HBV replication. Frozen and paraffin-embedded sections of liver biopsies from patients with CHB (Table 2 **)** were stained with a polyclonal galectin-9 antibody. We observed a high intensity of pan-lobular galectin-9 staining in all the sections from 8 HBV infected individuals, localising in sinusoids with a pattern suggestive of Kupffer cell expression (Figure 4a). Three-colour immunoflourescence combining galectin-9 with CD68 confirmed co-localisation of the majority of the intense galectin-9 staining with Kupffer cell (CD68) staining (Figure 4b). Expression of galectin-9 by CD14+CD68+ cells was further demonstrated by flow cytometry of freshly isolated Kupffer cells (Figure 4c).

Galectin-9 is a member of a large family of carbohydrate-binding lectins that lack signal sequences for conventional extracellular transport but are secreted by a non-classical pathway [1]. To assess whether HBV infection upregulates galectin-9, secreted levels circulating in patients with different levels of CHB disease activity were compared with those in healthy controls. There was a step-wise increase in circulating levels of galectin-9 according to disease activity (Figure 4d). Patients with biochemical evidence of highly active CHB-related liver disease (ALT>100 U/L) had significantly higher levels of serum galectin-9 than CHB patients with ALT<50 IU/L, (p = 0.01) or healthy controls (p = 0.02).

### Blocking Tim-3/galectin-9 Interactions Increases Functional HBV-specific T Cell Responses and is Complementary to PD-1 Blockade

To further define the contribution of Tim-3 expression to the dysfunction of HBV-specific T cells, we investigated the impact of blocking this pathway *in vitro*. To do this we used a recombinant Tim-3 Fc chimera to compete for galectin-9 and block the interaction of this ligand with Tim-3 expressed by T cells. Using this approach we were able to rescue additional CD8 T cells able to produce IFN-γ or TNF-α in response to stimulation with HBV peptides in 17 out of 32 (53%) and 17 out of 28 (64%) patients with CHB respectively (Figure 5). In 15/28 patients Tim-3 blockade resulted in the reconstitution of dual IFN-γ/TNF-α producing CD8 T cells (data not shown). Functional HBV-specific CD8 T cell responses could also be enhanced using a Tim-3-blocking mAb (kindly provided by V Kuchroo, Figure S2b).

Blockade of another co-inhibitory receptor, PD-1, has already been shown to rescue some HBV-specific CD8 T cell responses [8]. Tim-3-expressing cells were present in both the PD-1 positive and PD-1 negative HBV-specific CD8 T cell populations (Figure S3a), suggesting some overlap in the role of these co-inhibitory receptors. To explore whether PD-1 and Tim-3 played complementary roles in mediating T cell exhaustion in CHB we compared the effect of single or dual PD-1 and Tim-3 blockade on HBV-specific CD8 T cell responses. Virus-specific T cells were expanded with a pool of HBV peptides in the presence of Tim-3 Fc chimera, PDL1/2 blocking mAb, both or control IgG and analysed for the proportion of CD8 T cells producing IFN-γ and/or TNF-α (Figure 5a, 6a, b). Roughly equal proportions of the patient cohort responded to either of these approaches with the recovery of IFN-γ-producing CD8 T cells. Responders to Tim-3 or PD- L1/2 single blockade were largely not overlapping, suggesting non-redundant roles for Tim-3 and PD-1 (Figure 6a). Dual Tim-3 and PD- L1/2 blockade had an additive or synergistic effect on the recovery of HBV-specific CD8 T cells in some patients. This was most marked for TNF-α producing CD8 T cells, that were augmented by dual blockade in 12 out of 28 patients tested, significantly more than were rescued by either Tim-3 or PD-L1/2 blockade alone (Figure 6b, Figure S3b).

We then assessed the ability of Tim-3 blockade to enhance the expansion of CD8 T cells with cytotoxic potential by measuring their degranulation upon stimulation with HBV peptides. We found that 7 out of 8 patients showed enhanced cytolytic activity of HBV-specific CD8 T cells following Tim-3 blockade in culture (Figure S3c). In this smaller cohort, Tim-3 was more effective than PD- L1/2 blockade at rescuing cytolytic function of HBV-specific CD8 T cells (p<0.05, Figure 3d). The IL-2 producing capacity of CD8 T cells was also enhanced in 4 out of 9 patients (Figure S3e,f).

In a subset of 16 of the 28 patients in whom Tim-3 blockade was carried out, HBV responses were assessed using a pool of overlapping peptides spanning the HBV core protein, allowing the additional assessment of virus-specific CD4 responses (Figure S4a). A partial recovery of T cell function was also observed for CD4 T cells, with 11 out of 16 patients showing increased IFN-γ responses, 9 out of 16 increased TNF-α and 9 out of 9 increased IL-2 (Figure S4b,c,d). There was considerable overlap in responsiveness to Tim-3 and PD-1 blockade for CD4 T cells but five patients showed enhanced recovery of both IFN-γ and TNF-αCD4 responses upon dual compared to single blockade (Figure S5a, b). Of note, Tim-3 blockade alone was more effective at rescuing IL-2 producing CD4 responses than PD-1 blockade alone or in combination (Figure S5c, d).

### Tim-3 and PD-1 Contribute to Residual T Cell Exhaustion upon Potent Viral Load Reduction

Despite the ability of antiviral therapy to maintain viral suppression, this strategy alone is unable to reconstitute a durable antiviral T cell response [9], [15]. We investigated the role of Tim-3-mediated T cell co-inhibition in CHB patients starting antiviral therapy. In a cross sectional analysis we explored the expression of Tim-3 on global T cells from HBV infected individuals treated with antiviral therapy compared to our cohort of untreated patients. A non-significant trend to a lower frequency of Tim-3 expressing T cells was noted in patients on therapy (Figure 7a). To further explore this observation, we analysed 3 patients longitudinally from the time of initiation of antiviral therapy for more than one year into their antiviral regimen (Table 3). In two patients, Tim-3 expression increased on CD4 and CD8 T cells prior to starting treatment and did not fall below baseline levels on therapy, despite HBV DNA becoming undetectable. A third patient showed a progressive decrease in Tim-3 expression on CD4 and CD8 T cells accompanying the decline in viraemia seen upon initiation of antiviral therapy (Figure 7b). The incomplete reduction in Tim-3 expression is in line with the ongoing production of large amounts of sAg and persistent expression of other markers of T cell exhaustion in patients on antiviral treatment [9].

Having shown that Tim-3 can continue to be expressed on CD8 and CD4 T cells from individuals receiving antiviral treatment, we then examined whether HBV-specific T cells were still amenable to rescue by blocking Tim-3/galectin-9 interactions in vitro in these patients. CD8 T cells producing IFN-γ and/or TNF-α were still reconstituted by Tim-3 blockade in 2 out of 3 patients at the last on-treatment follow-up time point, when they had received at least 15 months of therapy. Similarly PD- L1/2 blockade continued to have an effect on CD8 T cell reconstitution in patients with effective viral load suppression. By contrast there was no further recovery of CD4 responses to Tim-3 blockade in 3 out of 3 patients and to PD- L1/2 blockade in 2 out of 3 patients by the last on-treatment follow-up time point (Figure 7c).

## Discussion

T cell responses are tightly regulated by multiple mechanisms in order to maintain homeostasis and prevent excessive inflammation within the immune system. Whilst these feedback mechanisms are important to allow termination of successful acute immune responses, they can excessively constrain antiviral immunity in the setting of persistent viral infections. This paradox is particularly evident in the liver, where the highly tolerising environment is likely to have evolved to preserve the functional integrity of this vital organ in the face of the high antigenic load it receives through the portal venous system [16]. Co-inhibitory pathways such as PD-1 are critical to hepatic tolerance and are further upregulated in viral hepatitis, contributing to the failure of T cell control of hepatic pathogens such as HBV and HCV. In this study we showed that co-inhibitory interactions between Tim-3 and galectin-9 are also activated in HBV infection and disable antiviral T cell immunity.

We found a higher expression of Tim-3 on global T cells, particularly the activated fraction, in patients with CHB compared to healthy controls and further enrichment of this co-inhibitory receptor within the liver-infiltrating fraction. Amongst circulating CD8 T cells, Tim-3 expression was significantly increased on the HBV-specific fraction compared to the generalised CD8 T cells or to CMV-specific CD8 T cells within the same patients. HBV-specific CD8 T cells expressing Tim-3 were associated with clinical outcome, since patients who had resolved HBV infection had significantly lower levels. Previous studies in patients persistently infected with HIV or HCV infection have shown that Tim-3 expression on T cells marks a highly dysfunctional subset of cells characterised by impaired production of antiviral cytokines such as IFN-γ and TNF-α and poor proliferative capacity. Similarly, we found that HBV-specific T cells that were unable to secrete these cytokines following short-term stimuli expressed higher levels of Tim-3. In addition to functional inactivation, interaction between Tim-3 and its ligand, galectin-9, may promote deletion of T cells, as evidenced by the induction of T cell death seen following this interaction *in vitro*. The ability of galectin-9 to trigger T cell death via Tim-3 is well-described, although the mechanisms remain poorly defined [17].

Whether the predominant effects of Tim-3/galectin-9 inteactions in patients with CHB are deletion or inactivation, the functional relevance of this ligand/receptor interaction is supported by the recovery of HBV-specific T cells with effector function following *in vitro* blockade. In more than half of our cohort of CHB subjects we were able to show some reversal of exhaustion in HBV-specific T cells, detecting an increase in cytokine producing cells following in vitro Tim-3 pathway blockade. We also observed an expansion of CD8 T cells with enhanced cytotoxic function when PBMC were co-cultured with anti-Tim-3 blocking agent, in line with recent findings in HCV infection [18]. The expansion of functional HBV-specific T cells in some, but not all, of our cohort supports the role of Tim-3 in T cell exhaustion, but also highlights the contribution of co-inhibitory receptors to their dysfunction [8], [9]. As reported previously [9], blockade of one or more inhibitory receptors can paradoxically diminish virus-specific responses in some cases, perhaps due to activation-induced cell death. The fact that Tim-3 blockade, like blockade of PD-1 or CTLA-4, cannot uniformly rescue responses is in line with the heterogeneity of HBV infection and the multiple layers of co-regulation characteristic of T cell exhaustion [19]. Our data showing a non-redundant role for Tim-3 and PD-1 in HBV are in line with previous reports showing that Tim-3 and PD-1 either mark distinct [6] or overlapping populations [5] of exhausted T cells in HIV and HCV infection respectively. A recent study of *in vivo* Tim-3/PD-1 blockade further demonstrated their synergistic cooperation in restraining T cell-mediated control of LCMV [20].

The ligand for Tim-3, galectin-9, is known to be a secreted protein, externalised by non-classical mechanisms [1]. In line with recent findings in HCV [21], we observed significantly elevated levels of galectin-9 in the circulation of those patients with HBV-related liver inflammation and also noted strong staining in Kupffer cells. A recent study has also reported galectin-9 expression on Kupffer cells (and weaker expression on dendritic cells) in the setting of hepatocellular carcinoma [22]. Kupffer cells are specialised, hepatic-resident macrophages that line the liver sinusoids. Galectin-9 expressing Kupffer cells are therefore ideally positioned to mediate deletion or functional inactivation of Tim-3 expressing T cells as they percolate slowly through the narrow and extensive intrahepatic circulatory bed. Systemic infusion of galectin-9 can inhibit the T cell responses driving immunopathology in herpes simplex virus infection [23]. Likewise, elevated levels of galectin-9 released in to the circulation of patients with CHB, perhaps as a feedback response to liver inflammation, could contribute to the maintenance of tolerance in T cells once they have left the galectin-9-rich environment of the liver sinusoids.

Galectin-9 can exert its inhibitory effect on T cells not only directly but also via the induction of other regulatory populations, including granulocytic myloid-derived suppressor cells MDSC [24] and Tregs [21], [23] that may expand preferentially because of their resistance to Tim-3-mediated apoptosis induction [23]. Future studies should therefore investigate whether galectin-9-expressing Kupffer cells can induce analogous regulatory populations augmenting the extrinsic regulation of antiviral immunity in the HBV-infected liver. Additional complexity has been added by the recent demonstration that Tim-3-galectin-9 interactions can also alter the function of the cells producing galectin-9; Tim-3-expresing T cells induced bactericidal activity in galectin-9 expressing macrophages infected with TB [25] and galectin-9 induced pro-inflammatory cytokine production by monocytes from HCV-infected livers [21]. Interventions to block the Tim-3 pathway could exert a potent reversal of the residual Tim-3-mediated immunosuppression that our data suggest persists in patients with CHB on antiviral treatment. However the potential application of Tim-3 blockade would need to take into consideration the bidrectional role of this pathway in regulating the balance between immunity and immunopathology and in promoting bacterial defense within macrophages.

## Materials and Methods

### Study Population

Clinical assessment and blood sampling were performed during routine hepatitis clinics, with written informed consent and local ethical board approval of the Royal Free Hospital, the Royal London Hospital and Camden Primary Care Ethics Review Boards. A total of 111 patients with CHB, 6 patients with resolved HBV and 20 healthy volunteers participated in the study; there were no significant differences in their demographics (Table 1). All the subjects were HCV and HIV seronegative. Patients with CHB were stratified by HBV DNA levels above or below 2,000 IU/mL (determined by real-time polymerase chain reaction [PCR]), according to European Association for the Study of the Liver guidelines [26]. Three patients with CHB were followed longitudinally after commencing lamivudine and adefovir (Table 3). Paired peripheral blood and liver biopsy specimens (surplus to diagnostic requirements) were obtained from eight patients with CHB (Table 2).

### Flow Cytometric Analysis of Total and Virus-specific CD8 and CD4 T Cells

PBMC were isolated from whole blood by Ficoll-Hypaque density gradient centrifugation; intrahepatic lymphocytes were isolated as previously described [9]. For analysis of total CD8 and CD4, cells were surface-stained with mAb anti-CD3 Pe-Cy7, CD8 Alexa700, CD4 APC-Cy7 (eBioscience), Tim-3 PE (R&D) or isotype matched control (R&D) in the presence of fixable live/dead stain (Invitrogen). In order to optimise the detection of low frequency HBV-specific T responses directly ex vivo, cells were stained with a combined pool of human leukocyte antigen A2 HBV dextramers (HLA-A2)/c18–27, HLA-A2/e183–191, HLA-A2/e335–343, HLA-A2/e348–357,HLA-A2/p455-) and HLA-A2/p502-) (Immudex). CMV-specific CD8 T cells were detected by HLA-A2/NLVPMVAYV pentamers (Proimmune) or A2/NLVPMVAYV dextramer (Immudex). Co-staining with Tim-3 mAb did not alter the percent of CD8 T cells able to bind dextramers (Table S1). B cells were stained with anti-CD19-V500 (ebioscience) and were excluded from the analysis of dextramer positive CD8 T cells. Where stated, virus-specific CD8 T cells expressing Tim-3 were stained with HBV dextramers before being stimulated with HBV peptides (10 µM) or control for 6 hours in the presence of 1 ug/ml Brefeldin A. For analysis of virus-specific T cells producing IFN-g and TNF-aor degranulation, the cells were first stained with CD107a PE and then permeabilised and fixed in one step (BD-cytofix ) followed by intracytokine staining with mAb anti-IFN-γ-APC (or Pacific blue) (eBioscience) and TNF-α-PE (BD).

Cells were acquired on a LSRII (BD Biosciences) and analyzed using Flowjo.

### Peptides

HLA-A2 negative patients were stimulated with a pool of 15mer peptides overlapping by 10 residues (OLP) spanning core of HBV genotype D. HLA-A2 positive individuals were stimulated with a panel of peptides representing immunodominant HLA-A2 restricted epitopes from HBV (envelope: FLLTRILTI, WLSLLVPFV, LLVPFVQWFV, GLSPTVWLSV; core: FLPSDFFPSV; polymerase: GLSRYVARL, KLHLYSHPI), CMV pp65 (NLVPMVATV).

### Assays of HBV-specific T Cell Function after *in vitro* Blockade

To examine the effect of blocking inhibitory pathways, PBMC were stimulated with or without HBV peptides (1 µM) in presence of Tim-3 FC chimera (R&D System) (2 µg/mL ), PD-L1, PD-L2 (eBioscience), or control IgG (BD-Biosciences) (5 µg/mL) or both Tim-3 FC chimera and PD-L1,PD-L2. Cells were cultured for 10 days, supplemented with 20 U/mL IL-2 at 0 and 4 days, restimulated at day 9 with 1 µM HBV peptide for 16 hours in the presence of 1 µg/mL Brefeldin A (Sigma-Aldrich), and identified by intracellular staining for IFN-γ, TNF-α and IL2. To assess degranulation function, CD107a antibody was added at the time of the restimulation at day 9.

### Flow Cytometric Analysis of Apoptosis and Cell Death

PBMC from patients with CHB were stimulated with 5 ug/ml recombinant galectin-9 for 6 hours at 37°C in the presence or absence of soluble Tim-3 FC chimera (R&D System) (2 µg/mL) or media alone. Carboxyfluorescein-FLICA (FAM-VAD-FMK) reagent was added during the last hour of culture. The degree of pancaspase activation was determined using the carboxyfluorescein-FLICA apoptosis detection kit (Serotec) according to the manufacturer’s protocol for detection by flow cytometry. Cells were also co-stained with anti-CD3 Pe-Cy7, CD8 Alexa700, and CD4 APC-Cy7 (eBioscience). 7-amino-actinomycin D (7-AAD) stain was used to label dead cells prior to acquisition.

### Galectin-9 ELISA

Serum samples (Table 2) were stored at −80. Plate ELISA assay for the detection of Galectin-9 was carried out according to manufacturer’s instructions (Uscn, Life Science Inc.).

### Immunohistochemistry and Immunofluorescence

Sections from paraffin-embedded and cryopreserved liver biopsies (Table 2) were stained for galectin-9. After being deparaffinized in xylene and rehydrated in a graded series of ethanol, paraffin embedded sections were rinsed in distilled water. An antigen retrieval step was performed by microwaving at 900W for 20 minutes in Tris EDTA buffer pH9. Alternatively cryopreserved HBV sections were air dried and washed in buffer prior to staining. Prior to incubation with the primary antibody, sections were blocked for endogenous peroxidase, washed gently in buffer, blocked with Serum Blocking Reagent and further treated with Avidin and Biotin Blocking Reagents (R&D Systems; Minneapolis, MN). The goat polyclonal galectin-9 antibody (R&D Systems; Minneapolis, MN) was used at a dilution of 1∶100 for frozen and 1∶300 for paraffin embedded biopsies for overnight incubation at 4°C. A Cell & Tissue Staining Goat Kit HRP DAB system (R&D Systems; Minneapolis, MN) was used for antibody detection. All staining steps were performed according to the manufacturers instructions. Visualization was achieved with DAB Chromogen solution followed by counterstaining with Mayer’s hematoxylin.

Immunostaining of cryopreserved sections from HBV infected liver biopsies was performed using a combination of the following primary antibodies Galectin-9 goat polyclonal IgG (R & D Systems) and CD68 mouse monoclonal (KP1, Abcam). Sections were incubated with Protein Block Serum Free (Dako) for 20 min at room temperature and then incubated overnight with primary antibodies diluted in PBS. Sections were then washed with PBS and stained with the appropriate secondary antibodies: Rabbit anti-goat Alexa Fluor 546 and donkey anti-mouse Alexa Fluor 488 (1∶500, Invitrogen) for 45 min at room temperature in the dark. Sections were washed and mounted with Vectashield mounting medium with DAPI (Vector Laboratories Inc). In the absence of a primary antibody no immunoreactivity was detected. Images were captured using a Zeiss Axioskop 2 plus microscope and AxioVision imaging.

### Kupffer Cell Isolation and Staining

Kupffer cells were isolated from liver sections as previously described [27]. Briefly, liver was digested using collagenase IV and DNase I, homogenised in a stomacher at low intensity and layered onto a density gradient (Optiprep 17%). The kupffer cell-rich fraction was plated onto a flat-bottom 6-well plate in DMEM with 10% FBS; non-adherent cells were washed off 2 hours post-plating. Adherent cells were trypsinised and co-stained *ex vivo* with mAb anti-CD14 V500 (BD) and Galectin-9 PE (Biolegend) or isotype matched control (Biolegend). Cells were then permeabilised and fixed in one step (BD-cytofix) followed by intracellular cytokine staining with mAb anti-CD68 FITC (eBioscience). Data was acquired on a LSRII (BD Biosciences) and analyzed using Flowjo.

### Statistical Methods

Mann Whitney test was used to compare differences between patient groups. Comparison between paired samples was carried out by Wilcoxon signed rank test. A P value of less than 0.05 was considered significant. Prism 4.0 software was used for all analysis.

## Supporting Information

Figure S1 **CD38 expression, intrahepatic Tim-3 expression and controls for multimer staining.** (a) Summary data showing CD38 expression on CD8 and CD4 T cells from CHB patients stratified according to their level of liver inflammation (ALT). **(**b) Representative FACS plots comparing Tim-3 expression on global CD8 (upper panel) and CD4 (lower panel) T cells from paired patient PBMC and intrahepatic lymphocyte (IHL) samples. Gating for Tim-3 was based on its isotype control for each sample. (c) Representative example of CD8 T cells stained with HBV multimers, a control multimer (bound with an irrelevant peptide) or no multimers directly *ex vivo* without stimulation (upper panel) or after peptide stimulation (lower panel).
(TIF)

Figure S2 **Effects of galectin-9 and sTim-3 Fc chimera and Tim3 blocking mAb.** (a) FACS plots showing the induction of caspases (FLICA) and 7AAD in CD8 and CD4 T cells with or without the addition of galectin-9+/− sTim-3 Fc chimera. (b) Representative dot plots comparing recovery of HBV-specific CD8 T cells responses (IFN-γ) with different Tim-3 blocking approaches in two patients with CHB. PBMC were cultured in media alone or stimulated with HBV peptides in the presence of control IgG, Tim-3 blocking mAb or soluble Tim-3 Fc chimera.
(TIF)

Figure S3 **Impact of Tim-3/PD-1 blockade on effector function of HBV-specific CD8.** (a) Summary data of the expression of Tim-3 on PD1+ and PD1- HBV-specific T cells. (b) Percent of HBV-specific CD8 T cells producing TNF-α following in vitro co-culture with HBV peptides and control IgG (white bars), soluble Tim-3 Fc chimera (red bars), PDL1/L2 blocking antibodies (green bars) and dual blockade (blue bars) in 28 CHB subjects studied (mean +−/SEM). Bar charts showing percent of CD8 T cells expressing CD107 (c) or IL-2 (e) after culture with HBV peptides in the presence of Tim-3 Fc chimera (red bars) compared to control IgG (white bars) in each patient with CHB. Background expression in unstimulated wells was subtracted. Summary data of percent of HBV-specific CD8 T cells expressing CD107 (d) or IL-2 (f) following in vitro co-culture with HBV peptides and control IgG (white bars), soluble Tim-3 Fc chimera (red bars), PDL1/L2 blocking antibodies (green bars) and dual blockade (blue bars).
(TIF)

Figure S4 **Blocking the Tim-3 pathway can increase the frequency of IFN-γ,TNF-α and IL2 producing HBV-specific CD4 T cells. (**a) Representative dot plots showing recovery of HBV-specific CD4 T cell responses (IFN-γ, TNF-α) following culture with HBV OLP in the presence of soluble control IgG, Tim-3 Fc chimera, PDL1&L2 blocking antibody or both for 10 days. Summary data on the effect of blocking Tim-3 (red bars) on IFN-γ (b), TNF-α (c) and IL-2 (d) production by CD4 T cells in response to HBV OLP compared to HBV stimuli without blockade (white bars). The background expression of IFN-γ, TNF-α and IL2 in unstimulated wells was subtracted.
(TIF)

Figure S5 **Comparison of Tim-3/PD-1/dual blockade on HBV-specific CD4 T cell effector function.** Summary data on the effect of blocking Tim-3 (red bars), PD-1 (green bars) or both pathways (blue bars) on IFN-γ (a), TNF-α (b) and IL2 (c) production by CD4 T cells in response to HBV OLP in patients with CHB. The bars represent the % of HBV-specific CD4 T cells producing cytokines following blockade after subtracting the frequency detectable without any blockade. (d) Summary bar charts of impact of blocking Tim-3, PD-1 or both on IL-2 production in response to HBV-specific peptides.
(TIF)

Table S1 **Frequency of HBV and CMV multimer staining cells (in the presence of Tim-3 or Tim-3 isotype mAb).**
(DOCX)
