# Supplementary figures and images for: Upregulation of the Tim-3/Galectin-9 Pathway of T Cell Exhaustion in Chronic Hepatitis B Virus Infection
Source: PLoS One. 2012 Oct 24;7(10):e47648. doi: 10.1371/journal.pone.0047648 (PMC3480425; doi:10.1371/journal.pone.0047648)

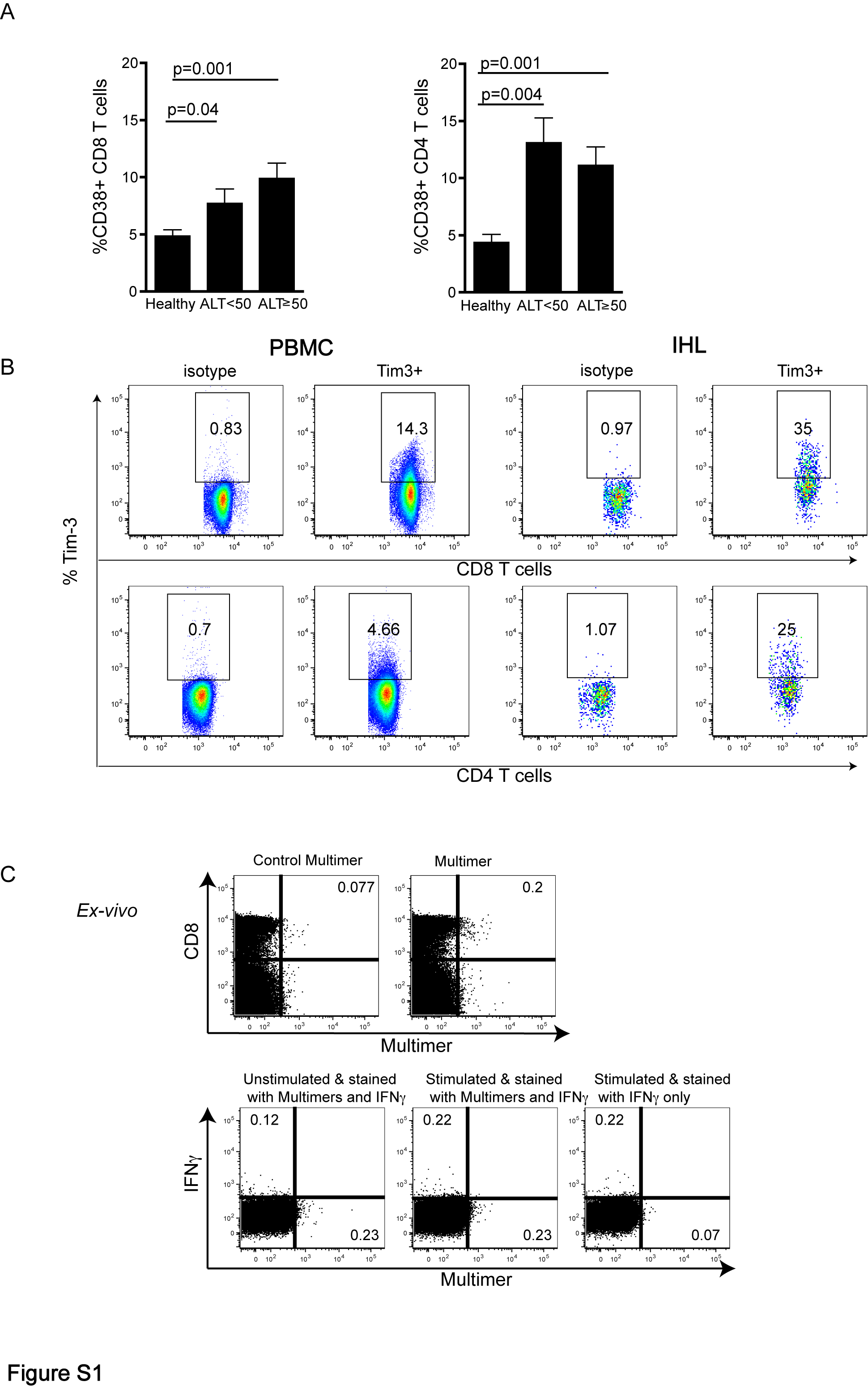

Supplement: Figure S1 — CD38 expression, intrahepatic Tim-3 expression and controls for multimer staining. (a) Summary data showing CD38 expression on CD8 and CD4 T cells from CHB patients stratified according to their level of liver inflammation (ALT). (b) Representative FACS plots comparing Tim-3 expression on global CD8 (upper panel) and CD4 (lower panel) T cells from paired patient PBMC and intrahepatic lymphocyte (IHL) samples. Gating for Tim-3 was based on its isotype control for each sample. (c) Representative example of CD8 T cells stained with HBV multimers, a control multimer (bound with an irrelevant peptide) or no multimers directly ex vivo without stimulation (upper panel) or after peptide stimulation (lower panel). (TIF) [file pone.0047648.s001.tif]

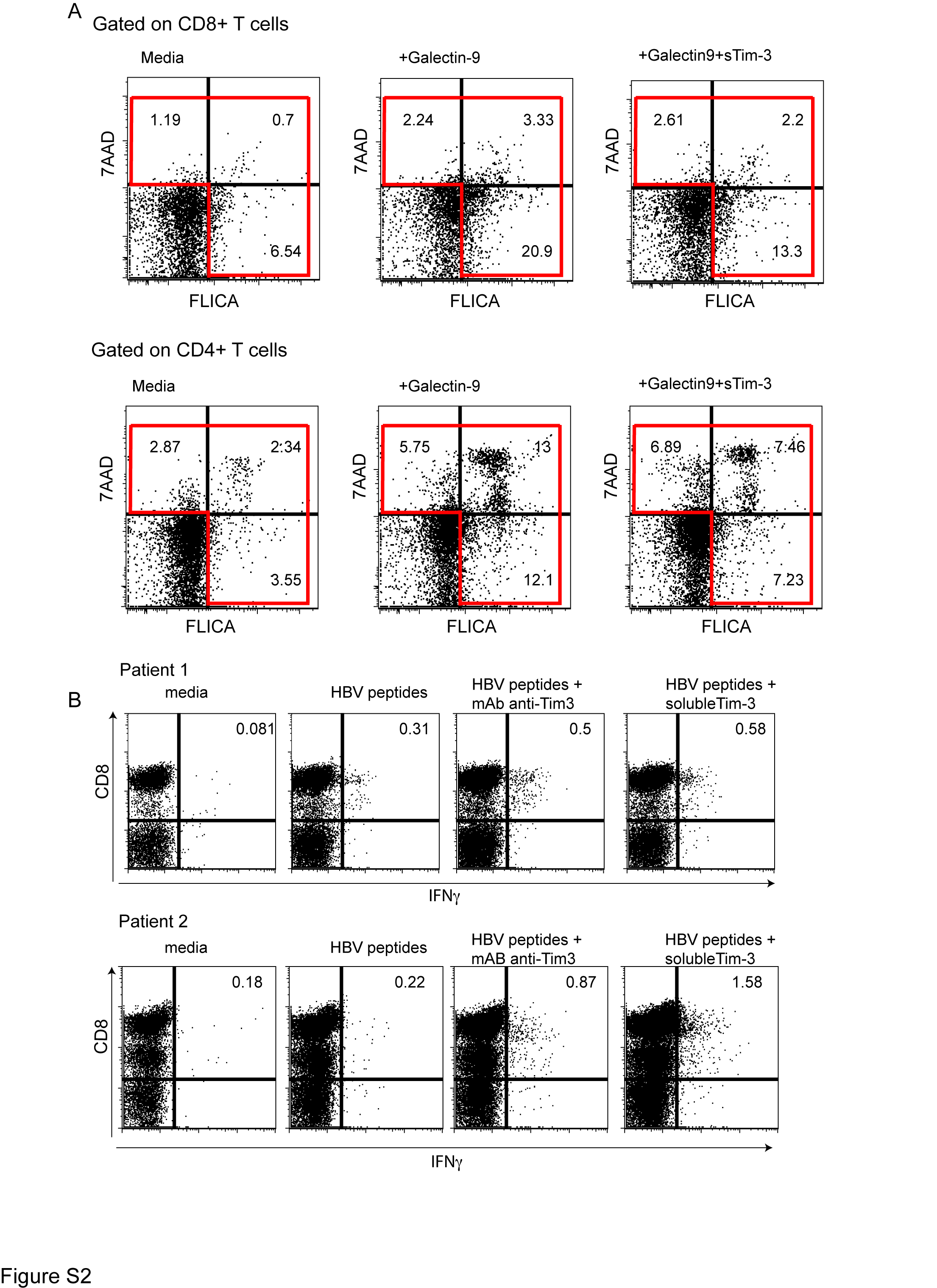

Supplement: Figure S2 — Effects of galectin-9 and sTim-3 Fc chimera and Tim3 blocking mAb. (a) FACS plots showing the induction of caspases (FLICA) and 7AAD in CD8 and CD4 T cells with or without the addition of galectin-9+/− sTim-3 Fc chimera. (b) Representative dot plots comparing recovery of HBV-specific CD8 T cells responses (IFN-γ) with different Tim-3 blocking approaches in two patients with CHB. PBMC were cultured in media alone or stimulated with HBV peptides in the presence of control IgG, Tim-3 blocking mAb or soluble Tim-3 Fc chimera. (TIF) [file pone.0047648.s002.tif]

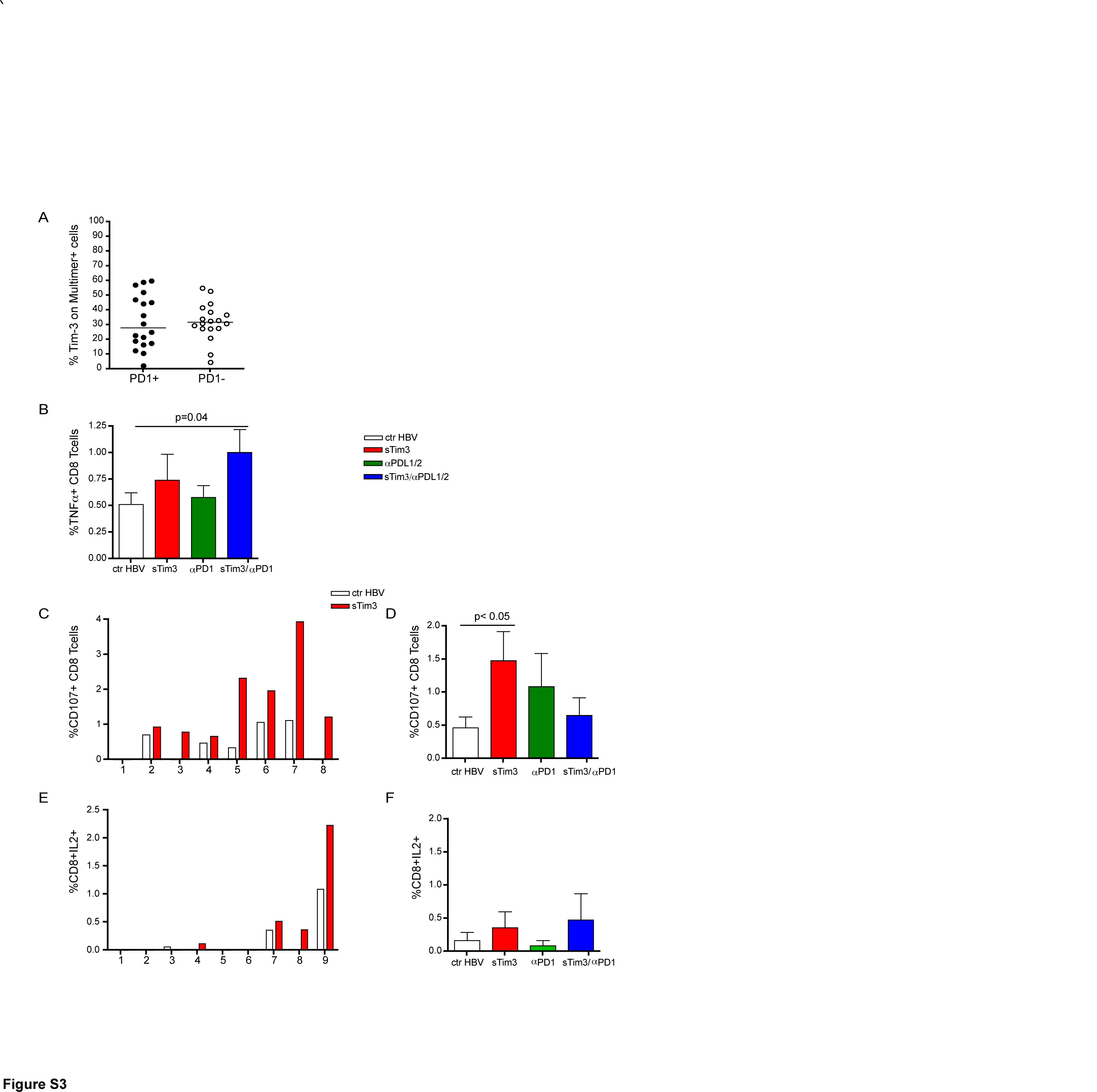

Supplement: Figure S3 — Impact of Tim-3/PD-1 blockade on effector function of HBV-specific CD8. (a) Summary data of the expression of Tim-3 on PD1+ and PD1- HBV-specific T cells. (b) Percent of HBV-specific CD8 T cells producing TNF-α following in vitro co-culture with HBV peptides and control IgG (white bars), soluble Tim-3 Fc chimera (red bars), PDL1/L2 blocking antibodies (green bars) and dual blockade (blue bars) in 28 CHB subjects studied (mean +−/SEM). Bar charts showing percent of CD8 T cells expressing CD107 (c) or IL-2 (e) after culture with HBV peptides in the presence of Tim-3 Fc chimera (red bars) compared to control IgG (white bars) in each patient with CHB. Background expression in unstimulated wells was subtracted. Summary data of percent of HBV-specific CD8 T cells expressing CD107 (d) or IL-2 (f) following in vitro co-culture with HBV peptides and control IgG (white bars), soluble Tim-3 Fc chimera (red bars), PDL1/L2 blocking antibodies (green bars) and dual blockade (blue bars). (TIF) [file pone.0047648.s003.tif]

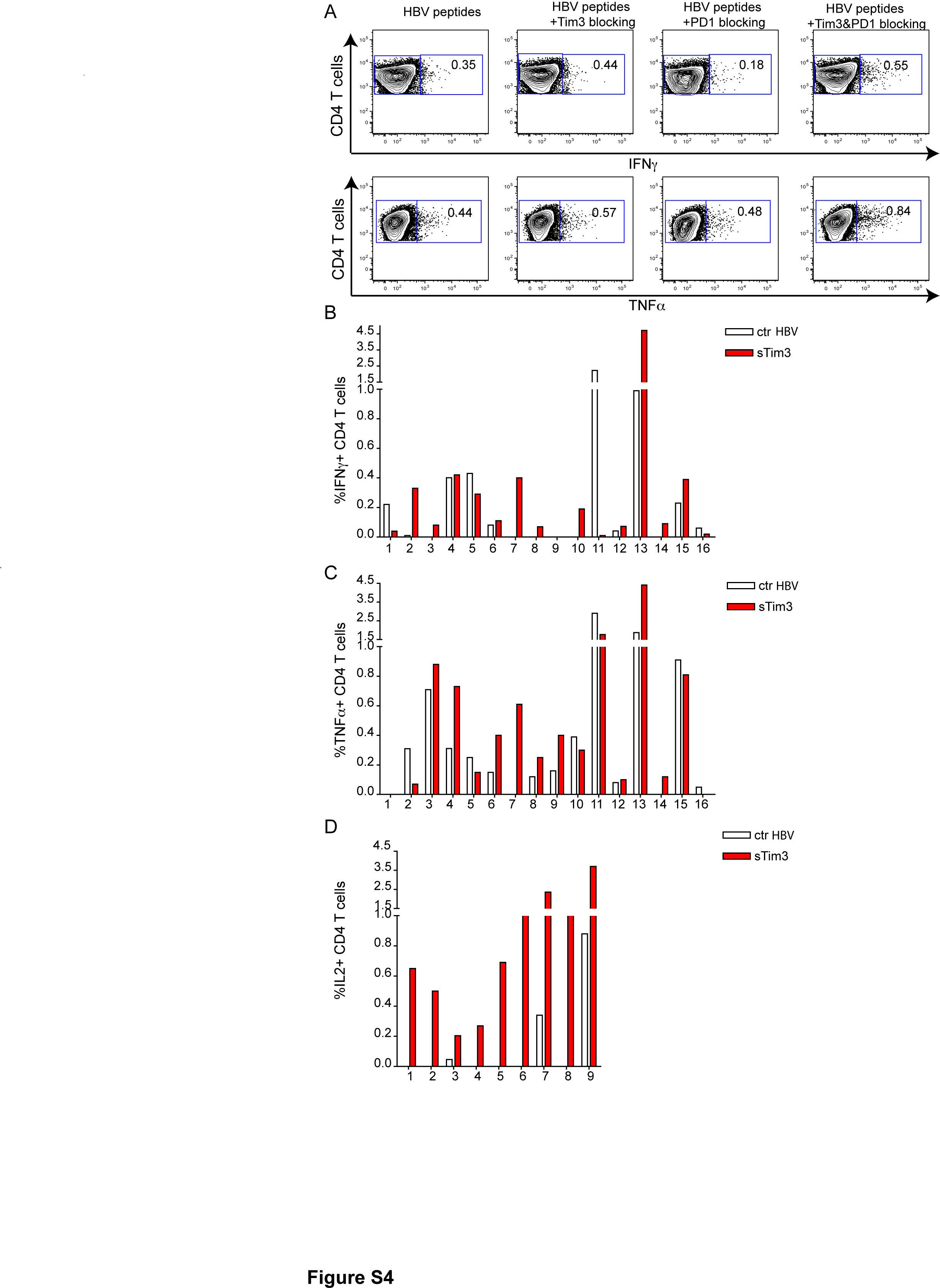

Supplement: Figure S4 — Blocking the Tim-3 pathway can increase the frequency of IFN-γ,TNF-α and IL2 producing HBV-specific CD4 T cells. (a) Representative dot plots showing recovery of HBV-specific CD4 T cell responses (IFN-γ, TNF-α) following culture with HBV OLP in the presence of soluble control IgG, Tim-3 Fc chimera, PDL1&L2 blocking antibody or both for 10 days. Summary data on the effect of blocking Tim-3 (red bars) on IFN-γ (b), TNF-α (c) and IL-2 (d) production by CD4 T cells in response to HBV OLP compared to HBV stimuli without blockade (white bars). The background expression of IFN-γ, TNF-α and IL2 in unstimulated wells was subtracted. (TIF) [file pone.0047648.s004.tif]

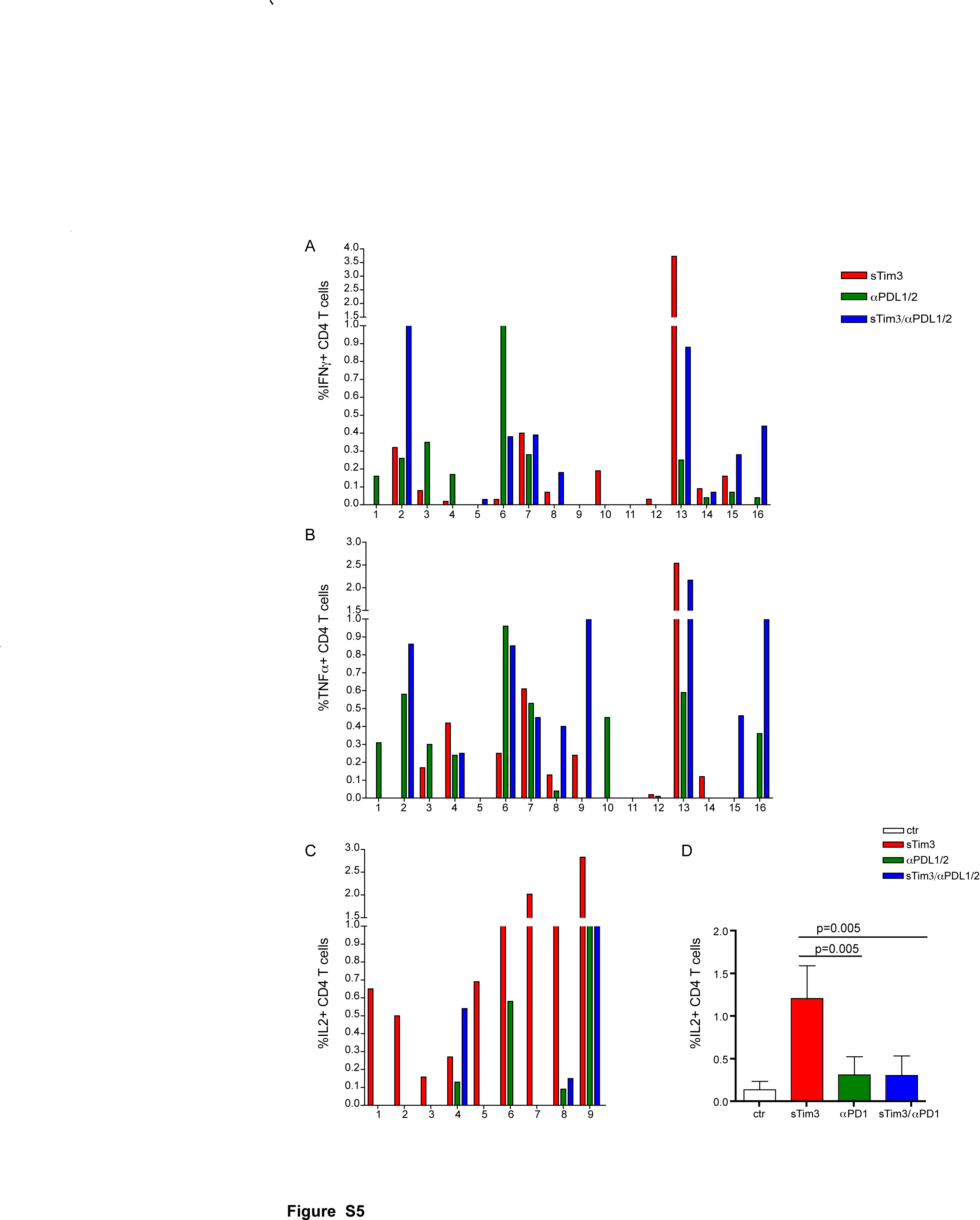

Supplement: Figure S5 — Comparison of Tim-3/PD-1/dual blockade on HBV-specific CD4 T cell effector function. Summary data on the effect of blocking Tim-3 (red bars), PD-1 (green bars) or both pathways (blue bars) on IFN-γ (a), TNF-α (b) and IL2 (c) production by CD4 T cells in response to HBV OLP in patients with CHB. The bars represent the % of HBV-specific CD4 T cells producing cytokines following blockade after subtracting the frequency detectable without any blockade. (d) Summary bar charts of impact of blocking Tim-3, PD-1 or both on IL-2 production in response to HBV-specific peptides. (TIF) [file pone.0047648.s005.tif]
