# Supplementary material for: Upregulation of the Tim-3/Galectin-9 Pathway of T Cell Exhaustion in Chronic Hepatitis B Virus Infection
Source: PLoS One. 2012 Oct 24;7(10):e47648. doi: 10.1371/journal.pone.0047648 (PMC3480425; doi:10.1371/journal.pone.0047648)
Supplement: Table S1 — Frequency of HBV and CMV multimer staining cells (in the presence of Tim-3 or Tim-3 isotype mAb). (DOCX) [file pone.0047648.s006.docx]

**Table S1:** Frequency of HBV and CMV multimer staining cells (in the presence of Tim-3 or Tim-3 isotype mAb).

|  | **% CMV-multime (with Tim-3 isotype)** | **% CMV-multimer (with Tim-3 mAb)** | **% HBV-multimer (with Tim-3 isotype)** | **% HBV-multimer (with Tim-3 mAb)** |
| --- | --- | --- | --- | --- |
| Patient 1 | 3.63 | 3.63 | 0.58 | 0.43 |
| Patient 2 | 0.22 | 0.18 | 0.62 | 0.68 |
| Patient 3 | 0.08 | 0.09 | 0.41 | 0.49 |
| Patient 4 | 0.22 | 0.3 | 0.71 | 0.69 |
| Patient 5 | 0.60 | 0.56 | 0.18 | 0.29 |
| Patient 6 | 0.08 | 0.09 | 0.51 | 0.52 |
| Patient 7 | 0.21 | 0.22 | 0.49 | 0.58 |
| Patient 8 | 0.16 | 0.17 | 0.63 | 0.59 |
| Patient 9 | 0.12 | 0.16 | 0.69 | 0.75 |
| Patient 10 | 0.22 | 0.3 | 0.15 | 0.13 |
| Patient 11 | 0.88 | 0.92 | 0.07 | 0.09 |
